# The microRNA miR-34a Inhibits Non-Small Cell Lung Cancer (NSCLC) Growth and the CD44^hi^ Stem-Like NSCLC Cells

**DOI:** 10.1371/journal.pone.0090022

**Published:** 2014-03-04

**Authors:** Yang Shi, Can Liu, Xin Liu, Dean G. Tang, Junchen Wang

**Affiliations:** 1 Department of Pathology, Shanghai East Hospital, Tongji University School of Medicine, Shanghai, China; 2 Department of Molecular Carcinogenesis, The University of Texas M.D. Anderson Cancer Center, Science Park, Smithville, Texas, United States of America; 3 Cancer Stem Cell Institute, Research Center for Translational Medicine, Shanghai East Hospital, Tongji University School of Medicine, Shanghai, China; Roswell Park Cancer Institute, United States of America

## Abstract

Lung cancer is among the most lethal malignancies with a high metastasis and recurrence rate, which is probably due to the existence of lung cancer stem cells (CSCs). CSCs in many tumors including non-small cell lung cancer (NSCLC) have been identified using adhesion molecular CD44, either individually or in combination with other marker(s). MicroRNAs (miRNAs) regulate both normal stem cells and CSCs and dysregulation of miRNAs has been implicated in tumorigenesis. Recently, miR-34a was found to be downregulated in NSCLC cells but the biological functions of miR-34a in regulating NSCLC cell behavior have not been extensively studied. Here we show that transfection of synthetic miR-34a, but not the negative control (NC) miRNA oligonucleotides (oligos) in three NSCLC cell lines, i.e., A549, H460, and H1299, inhibited their holoclone formation, clonogenic expansion, and tumor regeneration in vivo. Furthermore, the lentiviral vector-mediated overexpression of miR-34a in purified CD44^hi^ H460 cells also inhibited tumor outgrowth. In contrast, expression of miR-34a antagomirs (i.e., antisense oligos) in the CD44^lo^ H460 cells promoted tumor development. Our study shows that miR-34a is a negative regulator of the tumorigenic properties of NSCLC cells and CD44^hi^ lung CSCs, and establishes a strong rationale for developing miR-34a as a novel therapeutic agent against NSCLC.

## Introduction

Cancer stem cells (CSC), i.e., cancer cells with certain stem cell properties, have been reported in many human tumors and are thought to be responsible for tumor initiation, therapy resistance, progression, relapse, and metastasis [1]–[3]. MicroRNAs (miRNAs), small noncoding RNAs, regulate about 20%–30% of the genes in the human genome, and have been implicated in the regulation of proliferation, differentiation, migration, and apoptosis through inhibiting protein translation and/or inducing messenger degradation by binding to the complementary sequences of the 3′-untranslated region (3′-UTR) in their target mRNAs [4], [5]. miRNAs can act as both oncogenes and tumor suppressor genes [6], [7], and have emerged as important regulators of CSCs as well.

The microRNA-34a (miR-34a) functions as a tumor suppressor [8] and is downregulated in some human cancers, including breast cancer [9], prostate cancer [10], osteosarcoma [11], and lung cancer [12], [13]. Lung cancer is the most lethal malignancy worldwide. Work in the past several years indicates that both small-cell (SCLC) and non-small cell (NSCLC) lung cancers contain CSCs [14], [15]. As in most other tumors, potential lung CSCs have been enriched and purified using functional assays [16]–[18] as well as cell surface markers such as CD133, CD34, CD90, and CD44 [3]. CD44 is a membrane-bound glycoprotein that mediates a complex range of functions. Some studies have shown that the CD44^+^ cells are enriched for tumor-propagating capacity and that CD44 is a potential CSC marker in NSCLC [19]. Liu *et al* have shown that miR-34a can inhibit prostate CSCs and metastasis by directly repressing CD44 [20]. Identification of CD44 as a direct and functionally relevant miR-34a target reveals a previously unappreciated signaling pathway [20].

Although there is evidence that miR-34a is reduced in NSCLC, the biological functions of this miRNA in NSCLC remain scantily investigated. In this study, using a variety of biological assays combined with extensive xenograft tumor experiments, we report that miR-34a negatively regulates the CSC-associated properties as well as tumor-initiating capacity of three NSCLC cells.

## Materials and Methods

### Animals and animal experiments

Immune-deficient NOD–SCID (non-obese diabetic severe combined immune deficient) mice were produced mostly from our own breeding colonies and maintained in standard conditions according to the institutional guidelines. All animal-related studies in this project have been approved by the M.D. Anderson Cancer Center IACUC (Institutional Animal Care and Use Committee; ACUF# 08-05-08132). The current research does not involve human subjects (i.e., living individuals or identifiable private information). All other studies presented herein were the investigator-initiated and did not require approval from other regulatory bodies.

### Cells and basic experimental procedures

The three human NSCLC cell lines (A549, H460, and H1299) were obtained from ATCC. All cells were maintained in media recommended by ATCC supplemented with 1% penicillin/streptomycin and 10% fetal bovine serum (FBS; Invitrogen-Life Technologies). Cells were incubated in a humidified incubator at 37°C supplied with 5% CO_2_. Cells were routinely maintained in 75 cm^2^ tissue culture flasks (Corning Incorporated, USA) and harvested using 0.05% trypsin. Most basic experimental procedures have been described in our earlier publications [20], [21].

### Transient transfection with synthetic oligonucleotides (oligos)

We transfected bulk cells or the purified CD44^+^ NSCLC cells with 33 nM of miR-34a or non-targeting negative control miRNA (miR-NC) oligos (Ambion, Austin, TX) by using Lipofectamine RNAiMAX (Invitrogen). Alternatively, we transfected the purified CD44^−^ NSCLC cells with 33 nM of anti-miR-34a (anti-34a) or anti-miR-NC (anti-NC) oligos (Ambion). We generally harvested the transfected cells for in vitro or in vivo studies after culturing for 48 h.

### Lentiviral-mediated overexpression of miR-34a

A lentiviral vector encoding pre-miR-34a (lenti-34a) and the control vector (lenti-ctl) were obtained from Systems Biosciences (SBI) [20]. Lentivirus was produced in 293FT packaging cells and titers determined for GFP using HT1080 cells. NSCLC cells were infected at an MOI of 25 in the presence of 8 µg/mL polybrene and harvested 48–72 h post-infection.

### Quantitative RT-PCR

Mature miRNA and CD44 mRNA levels were quantified using TaqMan MicroRNA Assays (Applied Biosystems) [20]. Briefly, total RNA was isolated using the mirVANA PARIS miRNA Isolation Kit (Ambion). Quantitative miRNA expression data were normalized to internal ‘housekeeping’ miRNAs, i.e., miR-24 and miR-103. Quantitative mRNA expression data were normalized to internal ‘housekeeping’ mRNA, i.e., GAPDH. Differences between the positive and corresponding negative populations, i.e., ddCt values, for each of the miRNAs or mRNAs were converted to percentage of expression using the formula 2^−ddCt^ [20].

### Clonal and clonogenic assays

For holoclone assays [22], we plated NSCLC cells at a clonal density (i.e., 50 cells/well) in a 6-well dish, counted the number of holoclones several days later, and presented the percentage of cells that established a holoclone as cloning efficiency. For clonogenic assays [20], firstly, we mixed methylcellulose (MC) with serum-free medium supplemented with 5 µg ml-1 insulin, 20 ng ml-1 EGF and 10 ng ml-1 bFGF (Sigma). Then we plated cells generally at 1,000 cells/well in MC mixture at 1∶10 ratio in 24-well ultra-low attachment (ULA) plates and enumerated colonies 2–3 weeks after plating. For all above experiments, we run a minimum of triplicate wells for each condition and repeated the experiments whenever feasible.

### Flow cytometry analysis and fluorescence-activated cell sorting (FACS)

Expression of CSC markers was evaluated by flow cytometry. Cells were stained live in the staining solution containing BSA and FITC-conjugated monoclonal anti-CD44 (clone#G44-26; BD Bioscience) or PE-conjugated monoclonal anti-CD133 (clone#AC133; Miltenyi Biotech) at the concentration recommended by the manufacturers. Corresponding isotype-matched mouse immunoglobulins were used as negative controls (BD Bioscience). At least 10,000 cells were analyzed. For cell sorting, labeling of cell surface markers was performed under sterilized conditions and cells were sorted by BD FACSVantage Cell sorter (BD Bioscience). The top 10% most brightly stained, and the bottom 10% most dimly-stained cells were selected as the positive and negative populations, respectively. Sorting purity of over 90% was ensured for further in vitro and in vivo experiments. All data were analyzed by the Flowjo software (version 7.6.1).

### Aldefluor assay

The Aldefluor Assay Kit (Aldagen, Inc. Durham, NC) was used to profile the aldehyde dehydrogenase (ALDH) activity in NSCLC cells [23]. Cells were incubated in Aldefluor assay buffer containing the ALDH protein substrate BODIPY-aminoacetaldehyde (BAAA) for 40 min at 37°C. Cells that could catalyze BAAA to its fluorescent product BODIPY-aminoacetate (BAA) were considered ALDH^+^. Sorting gates for FACS were drawn relative to cells' baseline fluorescence, which was determined by the addition of the ALDH specific inhibitor diethylaminobenzaldehyde (DEAB) during the incubation and DEAB-treated samples served as negative controls. Non-viable cells were identified by Propidium Iodide (PI) positivity. Cells were sorted by BD FACSVantage Cell sorter.

### Tumor transplantation experiments

Cells were injected in 60 µl of medium∶Matrigel mixture (1∶1) subcutaneously (s.c) into NOD/SCID mice (6–8 weeks old). We transfected bulk H460, A549, or H1299 cells with miR-34a or miR-NC oligos (33 nM). 48 h later, three different cell doses at 500,000, 50,000, or 5,000 were implanted. For H460 cells, additional 2 million cells each (n = 7) were implanted. In addition to oligo transfection, we also infected some cells with lenti-34a or lenti-ctl vectors (MOI 25) [20], and, 48 h later, three cell doses at 500,000, 50,000, 5,000 were implanted. Finally, we transfected purified CD44^lo^ H460 cells with anti-34 or anti-NC oligos (33 nM) and also infected purified CD44^hi^ H460 cells with lenti-34a or lenti-ctl vectors (MOI 25). 48 h later, cells at different doses were implanted. Tumor growth was monitored on weekly basis and individual tumor volumes were measured using a digital caliper and approximated according to the formula V = 1/2ab^2^ (a being the long diameter and b the short diameter of the tumor). At the end of experiments, mice were sacrificed and tumors were harvested, measured, and photographed.

### Statistical analysis

We used unpaired two-tailed Student's *t*-test to compare differences in cell numbers, cloning efficiency, tumor weights, and other related parameters. We employed Chi-square test to compare incidence and latency. We used ANOVA (F-test) to compare differences in multiple groups. In all these analyses, a *P*<0.05 was considered statistically significant.

## Results and Discussion

### miR-34a inhibits NSCLC cell holoclone formation and clonogenic capacity

**Figure 1:**
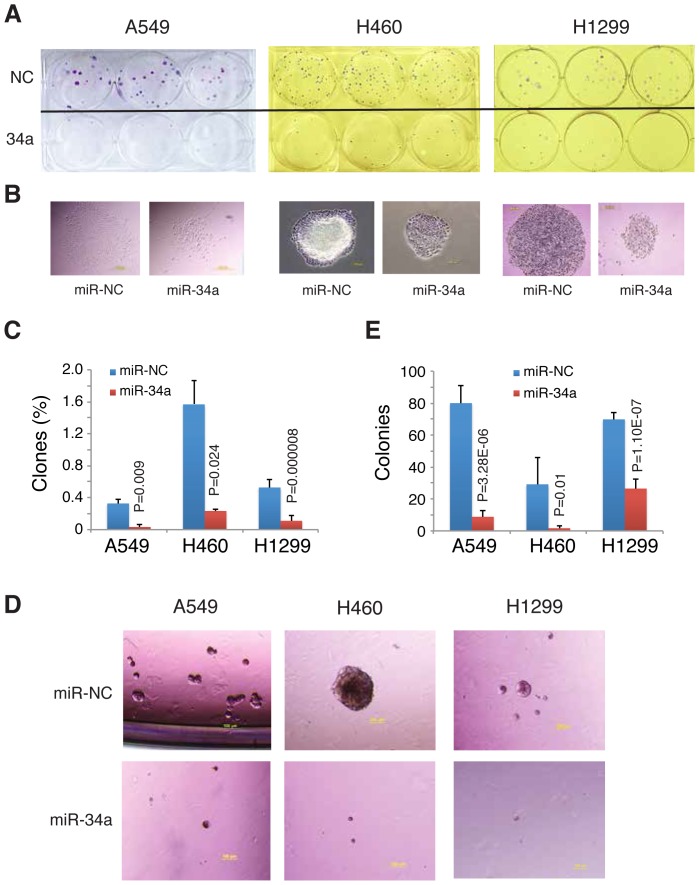
miR-34a inhibits NSCLC cell clonal and clonogenic properties. (**A–C**) Clonal assays. Cells transfected with miR-34a or miR-NC oligos (33 nM) were plated in triplicate at 50 cells/well in 6-well plates. The experiment was terminated at 12 d and wells were Giemsa-stained (A). Shown in B are representative images. Results shown in A and B were representative of two independent experiments. (C) Quantitative presentation of results in A. Bars represent the mean ± S.D. (**D–E**) Clonogenic assays in MC. A total of 1,000 cells per well were plated for clonogenic assay. Photos were taken on d 15 after plating and shown in D are representative fields. (E) Quantitative presentation of results in D. Bars represent the mean ± S.D.

miR-34a has been shown to possess tumor-suppressive functions [8], [20], [21], [24]–[33] and to be under-expressed in some tumors as well as certain tumorigenic subpopulations [10] such as CD44^+^ prostate CSCs [20]. miR-34a is a direct p53 target [32] and its promoter is silenced in some cancer cells [30]. There has been some experimental evidence that miR-34a is downregulated in NSCLC cells [12], [13]. To elucidate the potential biological consequence of loss of miR34a expression in NSCLC cells, we first transfected three NSCLC cells, A549 (p53 wild-type), H460 (p53 wild-type), and H1299 (p53 mutant), with synthetic mature miR-34a or miR-NC oligos (33 nM) [20] for 48 h, and then plated the cells in triplicate at clonal density (i.e., 50 cells/well) in 6-well plates. We then assessed the formation of holoclones, which have been shown to harbor self-renewing CSCs that can long-term propagate tumors [22], 12 days (d) after plating. As shown in Figure 1(A–C), miR-34a overexpression significantly inhibited holoclone establishment in all three NSCLC models. Of importance, although miR-NC transfected NSCLC cells formed large and tightly packed holoclones, the miR-34a transfected NSCLC cells founded much smaller and/or loosely packed paraclones (see Figure 1B for examples). Subsequently, we performed more stringent, anchorage-independent, clonogenic assays in methylcellulose (MC), which have been widely used to measure the cell-autonomous activity of potential CSCs [3]. As in clonal assays, miR-34a overexpression greatly inhibited the sphere-forming capacity of bulk A549, H460 and H1299 cells (Figure 1D–E).

**Figure 2:**
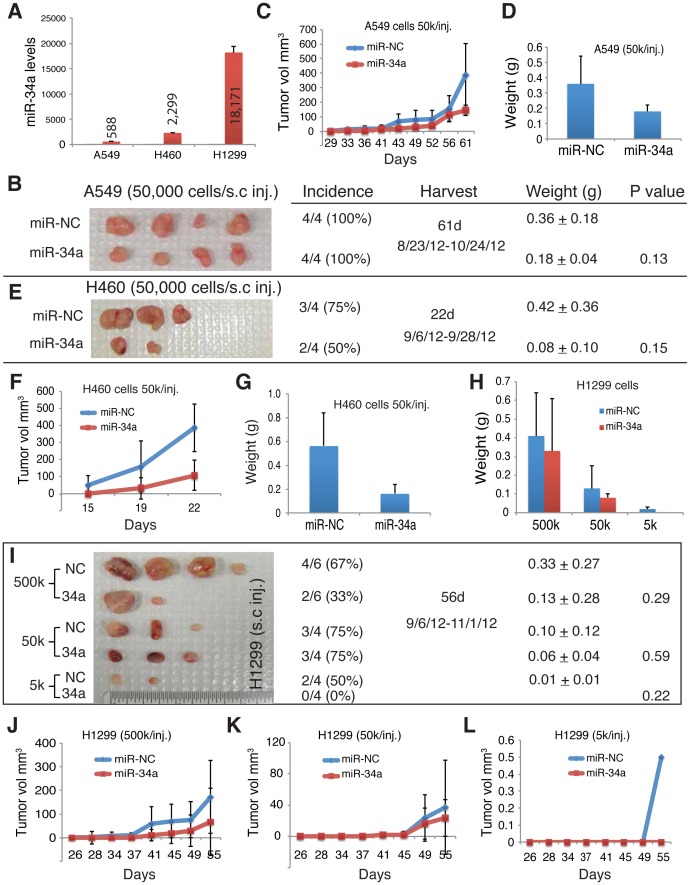
miR-34a inhibits NSCLC tumor growth. (**A**) NSCLC cells freshly transfected with miR-34a oligos showed miR-34a levels several orders of magnitude higher than those transfected with miR-NC oligos. The indicated NSCLC cells were transfected with miR-34a or miR-NC oligos, and 48 h later, were harvested and used in tumor experiments (below) whereas a small number of cells were set aside and used in qRT-PCR measurement of miR-34a mRNA levels. Shown are the mean miR-34a levels (in log scale; n = 2) in miR-34a transfected cells relative to those in the miR-NC transfected cells (actual mean values indicated in the bars). (**B–D**) miR-34a oligo transfection inhibited A549 tumor growth. Indicated are tumor incidence (tumors developed/numbers of injections; %), harvest time (including actual injection and termination dates), mean tumor weight (in grams), and the *P* values for tumor weights. Gross tumor images are not to the same scale. (**E–G**) miR-34a oligo transfection inhibited H460 tumor growth. (**H–L**) miR-34a oligo transfection inhibited H1299 tumor growth.

The above experimental results provide evidence that restoration of miR-34a expression in NSCLC cells inhibits both clonal and clonogenic properties. As the 3 NSCLC cells possess different p53 status, the results also suggest that the inhibitory effects of miR-34a are p53-independent. As expected, cells transfected with the miR-34a oligos showed dramatically increased miR-34a levels as assessed by qPCR analysis of mature miR-34a (Figure 2A). Interestingly, however, the 3 NSCLC cell types displayed a wide variation in the increased miR-34a levels, with H1299 cells retaining the highest amount of exogenous miR-34a 48 h after transfection (Figure 2A). As discussed earlier, miR-34a is a direct transcriptional target of p53 [32] and H1299 cells have mutant p53. One possibility is that both p53 and miR-34a levels in lung cancer cells must be very tightly controlled. Consequently, in A549 and H460 cells that have wild-type p53, even exogenously introduced p53 becomes degraded fast whereas in p53-mutant H1299 cells, the transfected mature miR-34a oligos survive much longer (Figure 2A).

### Evidence that miR-34a overexpression also inhibits tumor development

We then assessed the potential tumor-inhibitory effects of miR-34a on the three NSCLC cell types in vivo (Figure 2). We transfected 33 nM of miR-34a or miR-NC oligos into A549 cells for 48 hours and then implanted 50,000 (50 k) cells subcutaneously (s.c) into immune-deficient NOD/SCID mice. As shown in Figure 2B–D, miR-34a transfected cells developed tumors that were only about half the sizes of miR-NC tumors and the former also grew slower. It should be noted that the difference in tumor weights was not statistically significant due to the relatively small sample sizes. Similarly, miR-34a overexpressing H460 cells developed much smaller and slower-growing tumors compared to miR-NC transfected H460 cells (Figure 2E–G). H460 cells infected with a miR-34a lentiviral vector (i.e., lenti-34a) also regenerated smaller and slower growing tumors than the control vector infected cells (Figure S1A–C). Importantly, miR-34a overexpression in H460 cells reduced tumor incidence (75% in miR-NC vs. 50% in miR-34a, *P*<0.01; Figure 2E). Finally, we performed a limiting-dilution tumor assay by implanting 5 k, 50 k, or 500 k of miR-NC or miR-34a transfected H1299 cells in NOD/SCID mice and at every cell dose we observed smaller and slower growing tumors derived from the miR-34a overexpressing cells compared to the tumors from miR-NC transfected H1299 cells (Figure 2H–L). Again, miR-34a overexpression reduced tumor incidence at two cell doses implanted (i.e., 5 k and 500 k, *P*<0.01; Figure 2I). In fact, miR-34a transfected H1299 cells exhibited significantly lower tumor-initiating frequency (TIF) than corresponding miR-NC controls (*P* = 4.64E-179) as determined by using the Limdil function of the Statmod package (http://bioinf.wehi.edu.au/software/elda/index.html). The relatively stronger tumor-inhibitory effects of miR-34a on H1299 cells with respect to tumor incidence are likely related to much higher levels of exogenous miR-34a in transfected H1299 cells (Figure 2A).

As in the case of A549 cells, the differences in tumor weights in miR-34a vs. miR-NC transfected H460 and H1299 cells did not reach statistical significance, likely due to relatively small sample sizes (Figure 2E and 2I, respectively), which is a very common phenomenon in such xenograft tumor assays. Another plausible explanation is that the transfected oligos became gradually degraded in vivo, as we have repeatedly observed in similar tumor experiments using miR-34a [20] and let-7 [21] oligos. In support, the residual tumors derived from miR-34a transfected cells showed no increase in miR-34a (data was not shown) in contrast to freshly transfected cells (Figure 2A). One interesting observation was that the p53-mutant H1299 cells retained significantly higher levels of exogenous miR-34a (Figure 2A), which also seemed to manifest the strongest tumor-inhibitory effects in this cell line (compare Figure 2I vs. Figure 2B and 2E).

### miR-34a overexpression inhibits CD44^hi^ H460 tumor regeneration whereas anti-34a promotes tumor growth in purified CD44^lo^ H460 cells

**Figure 3:**
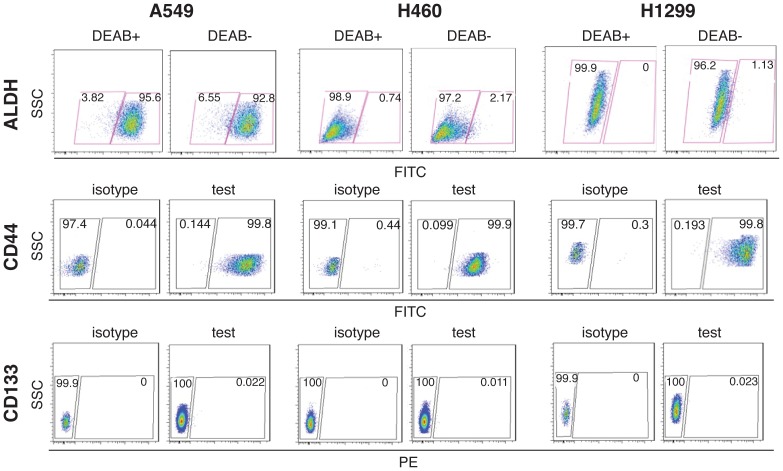
Functional assay (ALDH) and analysis of CD44 and CD133 expression using flow cytometry. (Top) The Aldefluor assay in 3 NSCLC cells. DEAB-treated samples served as negative controls. ∼1–2% H460 and H1299 cells were Aldefluor-positive whereas >90% of A549 cells were Aldefluor-positive. (Middle) Representative flow cytometry profile of CD44 (FITC) expression in 3 NSCLC cells. Virtually 100% of A549, H460, and H1299 cells were CD44-positive (mean values being 97.2%, 99.3%, and 99.2%, respectively; n = 3). (Bottom) Flow cytometry analysis of CD133 (PE) expression in 3NSCLC cells. There was almost no expression of CD133 in these three NSCLC cell lines.

There has been strong experimental evidence that miR-34a may manifest tumor-inhibitory effects by targeting CSCs [10], [20], [21] and NSCLC cell cultures have been shown to harbor stem-like cancer cells [3], [14]–[19]. Therefore, we wonder whether the biological effects of miR-34a on the 3 NSCLC cells (Figure 1 and 2) might be related to its action on stem-like cancer cells. To address this question, we first determined the percentage of cells positive for Aldefluor, CD44, and CD133, assays or markers frequently used to enrich lung CSCs. We observed ∼1–2% Aldefluor positive H460 and H1299 cells, which were largely ‘ablated’ in the presence of the ALDH inhibitor DEAB (Figure 3). For unknown reasons, several repeat experiments showed that >90% of A549 cells were Aldefluor-positive and this percentage was not affected by DEAB (Figure 3). Virtually 100% of A549, H460, and H1299 cells were CD44-positive (mean values being 97.2%, 99.3%, and 99.2%, respectively; n = 3) (Figure 3). In contrast, there was almost no expression of CD133 in these three NSCLC cell lines (Figure 3C). It is interesting that these assays might identify overlapping populations of stem-like NSCLC cells as purified ALDH^hi^ H1299 cells exhibited higher levels of CD44 mRNA (Figure S1D). In preliminary studies, we implanted 5 k or 10 k purified ALDH^hi^ and the corresponding ALDH^lo^ H1299 cells in NOD/SCID mice. At 5 k, the ALDH^hi^ and ALDH^lo^ cells developed 4/5 (1.22 g, 0.32 g, 0.24 g, and 0.12 g) and 1/7 (0.99 g) tumors, respectively. At 10 k, the ALDH^hi^ and ALDH^lo^ cells developed 3/8 (0.36 g, 0.2 g, 0.1 g) and 1/8 (0.03 g) tumors, respectively. These preliminary results suggest that the ALDH^hi^ H1299 cells possess higher tumor-regenerating capacity, an important CSC trait.

**Figure 4:**
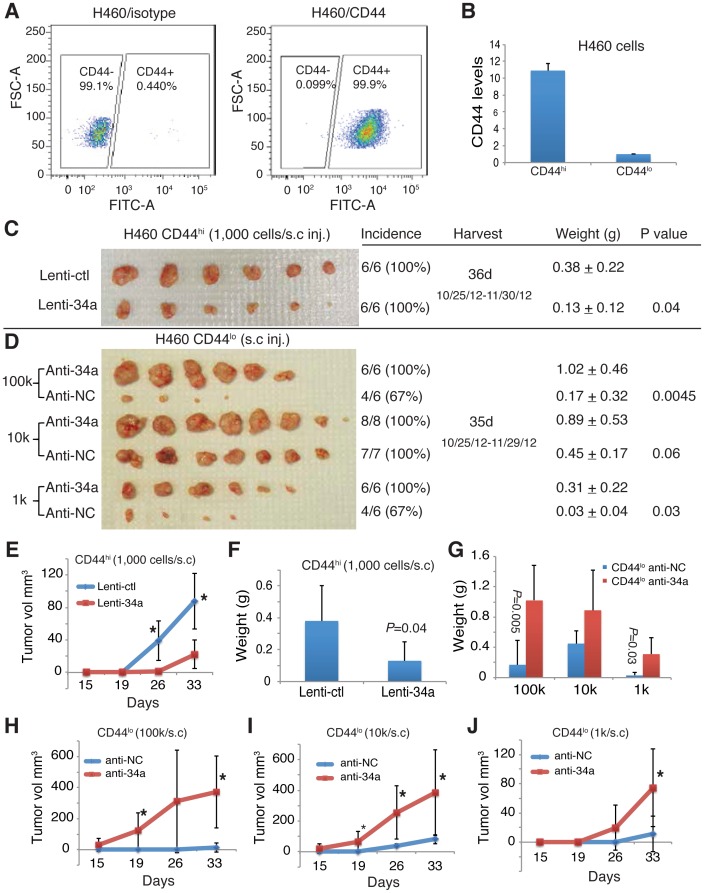
Effects of miR-34a on the growth of tumors derived from purified CD44^hi^ or CD44^lo^ cells. (**A–B**) CD44 expression level. (A) Representative diagrams of flow cytometry analysis of CD44 (FITC) expression in H460 cells. (B) CD44 mRNA levels in purified CD44^hi^ and CD44^lo^ H460 cells assessed by qRT-PCR. (**C, E, F**) miR-34a overexpression in purified CD44^hi^ H460 cells by lentiviral infection inhibited tumor regeneration. (C) Indicated are tumor incidence (tumors developed/numbers of injections; %), harvest time (including actual injection and termination dates), mean tumor weight (in grams, F), and the *P* values for tumor weights. Gross tumor images are not to the same scale. (E) The tumor growth curve. (**D, G–J**) Anti-miR-34a promoted tumor growth of purified CD44^lo^ H460 cells. (D) Indicated are tumor incidence (tumors developed/numbers of injections; %), harvest time (including actual injection and termination dates), mean tumor weight (in grams, G), and the *P* values for tumor weights. Gross tumor images are not to the same scale. (H–J) The tumor growth curve at three different cell doses.

In PC3 prostate cancer cells, nearly 100% cells are positive for CD44 [34]. However, there are PC3 cells that express very high levels of cell surface CD44 (i.e., CD44^hi^) and those low levels of CD44 (i.e., CD44^lo^). Of importance, CD44^hi^ PC3 cells demonstrated significantly higher clonal and clonogenic potentials than the isogenic CD44^lo^ PC3 cells [34]. Drawing on this analogy, we purified out CD44^hi^ (i.e., top 10%) and CD44^lo^ (i.e., bottom 10%) H460 cells (Figure 4A). As expected, the CD44^hi^ H460 cells expressed higher levels of CD44 mRNA than the CD44^lo^ H460 cells (*P* = 0.0009) (Figure 4B). Remarkably, lentiviral-mediated miR-34a overexpression in the CD44^hi^ H460 cells significantly inhibited tumor growth (Figure 4C,E, and F). More impressively, the anti-miR-34a antagomirs [20] dramatically promoted the tumor regeneration and tumor growth rate of CD44^lo^ H460 cells at all three cell doses (100 k, 10 k, and 1 k) tested (Figure 4D, G–J).

In summary, we have demonstrated that miR-34a overexpression inhibits NSCLC cell holoclone formation and clonogenic expansion in vitro and, importantly, tumor regeneration in vivo. These inhibitory effects of miR-34a might be due to its effects on stem-like NSCLC cells. In support of this possibility, enforced expression of miR-34a specifically in CD44^hi^ H460 cells greatly inhibited their tumor-regenerating activity whereas antagonists of miR-34a dramatically promoted tumor regeneration in CD44^lo^ H460 cells. These observations are consistent with CD44 being a direct and functional target of miR-34a in prostate [20] and some other cancer [31] cells and suggest that miR-34a negatively regulates the tumor-initiating capacity of NSCLC CSCs. Future work will aim to elucidate the underlying mechanisms and interactions between miR-34a and CD44 expression in NSCLC cells.

## Supporting Information

Figure S1 **miR-34a inhibits H460 tumor growth and ALDH^hi^ H1299 cells express higher CD44 mRNA levels.** (A–C) Lentiviral-mediated miR-34a overexpression in H460 cells inhibited tumor growth. (A) The endpoint tumor weights. (B and C) Tumor growth curves at two different cell doses. (D) The CD44 mRNA levels in purified ALDH^hi^ and corresponding ALDH^lo^ H1299 cells assessed by qRT-PCR.
(TIF)
