# Supplementary figures and images for: The microRNA miR-34a Inhibits Non-Small Cell Lung Cancer (NSCLC) Growth and the CD44hi Stem-Like NSCLC Cells
Source: PLoS One. 2014 Mar 4;9(3):e90022. doi: 10.1371/journal.pone.0090022 (PMC3942411; doi:10.1371/journal.pone.0090022)

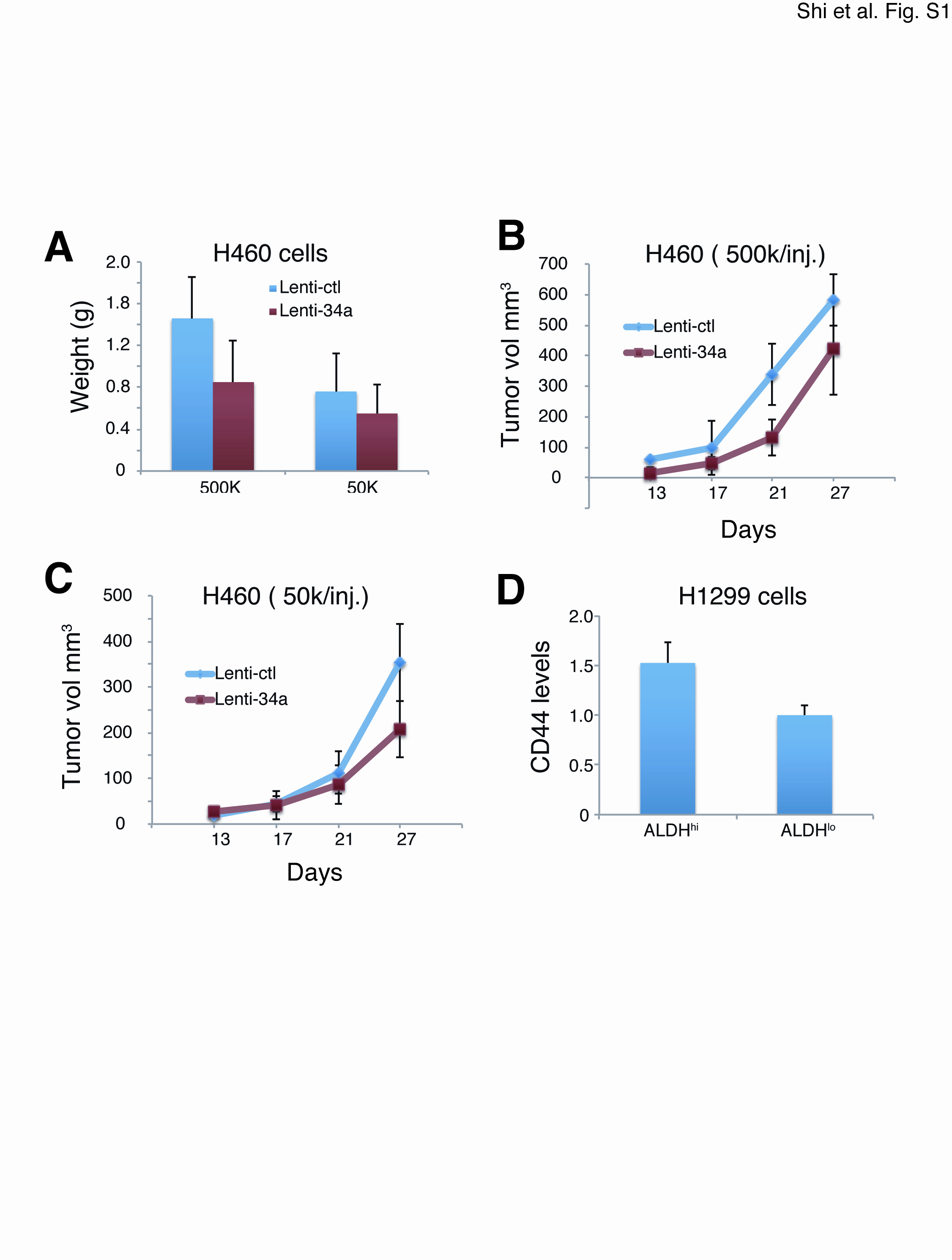

Supplement: Figure S1 — miR-34a inhibits H460 tumor growth and ALDHhi H1299 cells express higher CD44 mRNA levels. (A–C) Lentiviral-mediated miR-34a overexpression in H460 cells inhibited tumor growth. (A) The endpoint tumor weights. (B and C) Tumor growth curves at two different cell doses. (D) The CD44 mRNA levels in purified ALDHhi and corresponding ALDHlo H1299 cells assessed by qRT-PCR. (TIF) [file pone.0090022.s001.tif]
